# The effect of cash transfers on mental health – new evidence from South Africa

**DOI:** 10.1186/s12889-020-08596-7

**Published:** 2020-04-03

**Authors:** Julius Ohrnberger, Eleonora Fichera, Matt Sutton, Laura Anselmi

**Affiliations:** 1 Department of Infectious Disease Epidemiology, Faculty of Medicine, Imperial College London, School of Public Health, 47 Praed Street, St Mary’s Campus, London, UK; 2 Department of Economics, University of Bath, Bath, UK; 3 Division of Population Health, Health Services Research & Primary Care, University of Manchester, Manchester, UK

**Keywords:** Mental health, Poverty, Cash transfer, Instrumental variable estimation, Fixed effects, South Africa

## Abstract

**Background:**

Mental health and poverty are strongly interlinked. There is a gap in the literature on the effects of poverty alleviation programmes on mental health. We aim to fill this gap by studying the effect of an exogenous income shock generated by the Child Support Grant, South Africa’s largest Unconditional Cash Transfer (UCT) programme, on mental health.

**Methods:**

We use biennial data on 10,925 individuals from the National Income Dynamics Study between 2008 and 2014. We exploit the programme’s eligibility criteria to estimate instrumental variable Fixed Effects models.

**Results:**

We find that receiving the Child Support Grant improves adult mental health by 0.822 points (on a 0–30 scale), 4.1% of the sample mean.

**Conclusion:**

Our findings show that UCT programmes have strong mental health benefits for the poor adult population.

## Background

About 1.1bn people worldwide suffer from mental health problems. There is evidence of a strong relationship between poor mental health and poverty [28], which stands in line with the social causation hypothesis that adverse socio-economic factors precede and cause mental health problems [9]. The link between poverty and mental health makes mental health a global development problem [36]. As unconditional cash transfer (UCT) programmes are used to alleviate poverty, it is natural to investigate their effect on mental health.

One can use the Grossman model of health capital [17] to hypothesise different possible effects of UCTs on mental health. Health has a dual nature in the model. It is a consumption good as it directly generates utility, but also an investment good (human capital), required for income generating activities. Based on the Grossman model, the effect of UCTs on mental health can be positive, negative, or null. The effect can be positive, as UCTs can increase household income and thus directly increase mental health investment opportunities or reduce the opportunity cost of time for mental health investment activities. Releasing the financial constraint can have an immediate direct effect on the psychological wellbeing of household members by giving (more) financial security [27]. However, an increase in income can also potentially lead to worse mental health outcomes through increased consumption of unhealthy goods such as alcohol and consequent worsening of and mental health in the long run [14]. They can be ineffective as the monetary assistance provided does not enforce behavioural changes, give information or encourage investments in mental health.

A recent systematic review of the effects of cash transfers on social determinants of health in the Sub-Saharan Africa highlighted that cash transfers, both conditional and unconditional, have shown potential to improve mental health [33]. However, there is limited and mixed evidence on the quantitative effects of UCTs on mental health. Two studies from South America have found no significant effects of UCTs on mental health [13, 34], four studies from Sub-Saharan Africa identified positive effects on mental health [5, 11, 13, 20], whereas one study from Sub-Saharan Africa identified only significant effects for male adolescents but not female adolescents [22].

Existing studies focus on small samples of females or adolescents and fail to identify the population-wide effects of UCTs on mental health. They are either cross-sectional or short-term analyses and do not to provide information on the long-term effects of UCTs on mental health. Both pieces of information are important for policy makers aiming at sustainably improve mental health in LMICs [30]. Our research aims to fill these gaps in the literature.

We contribute to the literature by estimating the effects of the South African Child Support Grant (CSG) UCT programme on the mental health of a representative sample of the poor adult population from South Africa. We use as measure for mental health the Centre for Epidemiological Depression Scale (CES-D). We further extend the literature by using longitudinal data covering information about individuals up to 6 years which is unique in the existing literature. The CSG is South Africa’s largest social cash transfer programme and a long-term UCT programme. We focus on South Africa in the analysis for the high prevalence of mental health disorders in the country, with one in six of the population suffering from depression or anxiety [35].Unipolar depression has the highest prevalence of all mental illnesses among the South African population contributing to 5.8% of the overall burden of disease, which is 1.5 times higher compared to other Low- and Middle-Income Countries (LMICs) [21].

We use data from four waves (2008–2014) of the South African National Income Dynamics Study (NIDS) on 10,925 individuals living in poor households. We address selection into the CSG programme using eligibility as an instrument for grant receipt and account for unobserved individual heterogeneity in mental health by using Fixed Effects models. We find that the UCT programme improves adult mental health by 0.822 points (on a 0–30 scale) corresponding to 4.1% of the sample mean. The CSG effect on mental health is heterogeneous by gender, with only significant effects for females. Our study is the first to provide evidence on the effect of UCT programmes on adult mental health in general, showing that UCT can have strong positive effects.

The paper is structured as follows: the first section presents the study background, the second section describes the UCT programme, the third section describes the data, the fourth section describes the methods, the fifth section presents the descriptive statistics and results, and the sixth section discusses the results and concludes.

## The child support Grant (CSG) programme

The Child Support Grant (CSG) was introduced by the South African Government in 1998 as part of the governmental social assistance programme [39]. The CSG is a UCT and the South Africa’s largest social cash transfer programme, targeting the poor and vulnerable population [16]. The CSG aim is to reduce poverty and vulnerability among children from poor socio-economic backgrounds by monthly grant transfers to their primary caregivers, which in most cases is the biological mother of the child [11].

Eligibility for programme participation is defined by two factors: (1) the age of the child; and (2) a means-test of the caregiver and his/her spouse’s income and assets. The primary caregiver is defined as the main responsible person for the child and his/her daily needs [35]. The cash transfer is substantial for poor households, as it increases household income by about 20–25% [16]. The coverage of the CSG increased over time [16], as illustrated in Table A1 in the Additional file 1. Initially, only children up until the age of 7 years were targeted, but the programme has been progressively extended over time to children of age up to 18 years. The income threshold was also lifted at a faster rate than price-inflation, from R2,300 (US$172.5) to R3,200 (US$240), leading to a positive real appreciation of the grant value.

This makes the CSG a suitable cash transfer programme to identify the average effects of unconditional cash transfers on the poor adult population. The CSG represents a monetary shock to the average poor household in South Africa, which contains on average three to four children [44]. Additionally, evidence shows that although targeted at children, the transfers are shared at the household level [8, 10] raising the possibility of within household spill-over effects on all members.

The actual receipt of the CSG ultimately depends on eligible caregiver decisions as they must register. Selection into the programme is an important issue as children eligible and in need may miss out on the support. Recent studies show that about 27% of the eligible children have not received the grant [16]. We discuss the selection problem in more detail in section *5.3*.

## The National Income Dynamics Study (NIDS)

### Survey structure and coverage

We use four waves of data from the National Income Dynamics Study (NIDS; 2008–2014). The NIDS is a biennial longitudinal survey of a nationally representative sample of the South African population [40]. A two-stage cluster sample design was used [26]. Stratification was made at the district council level and clustering was implemented by primary sampling unit (geographical areas consisting of at least one enumeration area within the district council level). The first wave of the survey was conducted between January and December 2008 and consisted of 28,226 individuals (9605 children and 18,621 adults) from 7296 households. The same individuals were re-interviewed and new household members were included in the following waves. We use the adult sample of the survey which includes all individuals of age 15 years and above (average age 39) at the time of the survey interview. We include in our analysis only individuals that are observed at least twice across waves.

Across all waves we use the lower income groups of the NIDS defined by the 2008 CSG income-eligibility lower-upper bound threshold of R800 (US$60) per capita per month in 2008. This is the sample for which the CSG eligibility criteria are satisfied. In doing so, we use the sample of poor individuals, as the lower-upper CSG bound is close to the upper-bound of national poverty line in 2014 of R779 (US$58) [43]. The sample of individuals with an income below the upper poverty threshold in 2014 satisfies the means-test used for the CSG as the upper poverty threshold is always lower.

### Mental health measure

We measure mental health using a validated 10-item version of the Centre for Epidemiological Depression Scale (CES-D) scale developed by Radloff [37]. The CES-D scale is a continuous variable that is scored from 0 to 30 with higher scores implying worse mental health. However, for ease of interpretation, we invert the scale of the CES-D ranging from zero (high depression and worst mental health status) to 30 (no depression and best mental health status).

The CES-D is a robust and clinically validated measure for depression that has been widely applied in the analysis of cash transfer effects on mental health [13, 20, 22, 34]. It is based on measures self-reported by the participants and collected in every wave of the NIDS. The CES-D scale has been used in a wide set of longitudinal studies and is a validated measure for the poorer populations living in South Africa [6].

### Treatment variable and instrument

The explanatory variable of interest is a binary variable taking value one if an individual lives in a household in which at least one person receives the CSG grant and zero otherwise, and is based on individuals’ reports. We use a binary instrumental variable which takes value one if the individual lives in a household which has at least one child of eligible age to receive the CSG, and zero if the individual lives in a household without a CSG-age-eligible child. This age-eligibility binary variable has been used as an instrumental variable for CSG-receipt in previous research [12].

### Control variables

As mental health is influenced by life events [1], we add a binary variable indicating if the death of a household member had occurred in the past 2 years. We add a variable indicating the number of household members to control for social isolation, which is associated with poor mental health outcomes [1], and for differences in household size between CSG-receiving and non-receiving households [10]. Other variables commonly used to proxy social capital, such as social interaction or group membership, are not available throughout the waves. We include age to account for the association between age and mental health problems and age squared because of the inverted U-shape in the age distribution of wellbeing [7]. Individuals aged 15–18 years old, are potentially eligible to receive the CSG on their own behalf. To control for any variation in mental health for this specific group, we add a binary variable indicating if the individual is aged below 19 years. We include gender, as the literature shows differences in mental health and in cash transfer programme effects on mental health by gender [22].

We control for whether an individual is the economic decision maker responsible for investment and expenditure decisions of a household, as research has found a negative relationship between being responsible of economic decision making and perceived stress in low income settings [31].

At a community level, poor intangible assets are found to be associated with poor mental health [51], we control for neighbourhood effects using a categorical variable indicating the frequency of burglary in the neighbourhood. We include a set of binary variables indicating the level of common burglary and presence of theft in the neighbourhood of the respondent (none, very rare, not common, fairly common or very common) and use none as baseline.

Variations across the provinces of South Africa in terms of rurality and in terms of poverty are large and correlated with mental health [28, 43]. Moreover take-up of the CSG varies by provinces, with Gauteng and North West showing the highest exclusion of children from CSG receipt [47]. We use a set of province binary variables using Limpopo as baseline to control for the province of an individual (Limpopo, Western Cape, Eastern Cape, Northern Cape, for Free State, Kwa Zulu-West, North West, Gauteng, and Mpumalanga). We also include a set of binary variables to control for whether the individual lives in a formal rural setting, a tribal authority area, or an urban formal or urban informal (township) setting. Controlling for rural and informal urban areas is also important as CSG-receipt is lower in these areas where individuals are less likely to sign up due to poor representation and distance to local authorities and the increased cost of travel [10].

Individuals are also eligible to apply for other government grants alongside the CSG [39]. To control for any contamination effects of the other grants on the CSG effect on mental health, we add a set of four binary variables for whether the individual lives in a household that receives any of the other main support programmes (Foster Care Grant (FCG), the Disability Grant (DG), the Care Dependency Grant (CDG), or the Old Age Pension (OAP)). We further include a set of 19 binary variables of child age to all estimations. These 19 binary variables indicate if children of age [0, 18] live in the household of the respondent and consequently will pick up any possible variation of the instrumental variable with mental health induced age of the cohabiting children.

## Methods

### Pooled cross-sectional model estimation

In order to investigate the association between CSG household receipt and mental health, we estimate the following OLS model:

1 $$ CE{S}_{i,t}={\beta}_0+{\beta}_1{CSG}_{i,t}+{\boldsymbol{X}}_{i,t}{\beta}_2+{\boldsymbol{T}}_{\boldsymbol{i}}{\beta}_3+{\boldsymbol{R}}_{\boldsymbol{i}}{\beta}_4+{v}_{i,t,} $$

where *CES*_*i*, *t*_ is the CES-D score of the individual *i* in year *t*. The CES-D score was assessed in all four waves of the NIDS. *CSG*_*i*, *t*_ is a binary variable indicating if an individual lives in a CSG-receiving household. **X** is a vector of control variables which includes, ***T***_***i***_ is a set of year dummies to control for time trend effects and ***R***_***i***_ is a set of regional dummy variables to control for regional variations.

We cluster standard errors on the primary sampling unit in all models because common cluster effects may occur at local level [49]. We also test for potential attrition effects in all models by including a dummy variable taking on a value of one if the individual left the survey due to death or other reasons in the following wave and zero otherwise [48]. We provide an analysis of sample attrition and sample transition (leaving the “poor” sample or moving into the “poor” sample) in section *A3* of the Additional file 1.

### Fixed effect estimation

The OLS estimation could be downwardly biased because of potential unobserved individual heterogeneity in mental health driven by unobservable behaviours and preferences [19]. We account for unobserved individual time-invariant heterogeneity in mental health using Fixed Effects (FE). By doing so, we focus on variation within individuals over time. FE address the correlation of the beta-coefficient with the unobserved individual component of the error term *v*_*it*_ = *a*_*i*_ + *u*_*i*, *t*_. *a*_*i*_ is the unobservable time-invariant component and *u*_*i*, *t*_ is the time-varying component, uncorrelated with the covariates, of the individual error term. FE account for time invariant determinants such as traumatic events, adverse childhood events or genetic health endowments which are important in shaping mental health but are not observed [15]. Therefore, we formally estimate:

2 $$ CE{S}_{i,t}={a}_0+{a}_1{CSG}_{i,t}+{\boldsymbol{X}}_{i,t}{\alpha}_2+{\boldsymbol{T}}_{\boldsymbol{i}}{\alpha}_3+{\boldsymbol{R}}_{\boldsymbol{i}}{\alpha}_4+{a}_i+{u}_{i,t} $$

### Instrumental variable (IV) estimation

Selection into the CSG programme can occur for several reasons: exclusion errors occur due to lack of information about the programme, incomplete documents, distance to local authorities and related opportunity costs and travel costs, or the lack of proof of eligibility of the child’s age and identity [8, 10, 16]. Furthermore, individuals with poorer mental health outcomes could be less likely to apply for the grant. Therefore, omitted variable bias can affect the estimate of the effect on mental health. Selection bias could downwardly bias the estimated treatment effects.

We address selection into the CSG programme by using an instrumental variable approach, which is an appropriate method to deal with selection due to unobservable factors [4]. We instrument the binary variable of living in a CSG receiving household with a binary measure taking value one if at least one child eligible for the CSG grant lives in the household and zero otherwise. Our instrumental variable satisfies the two criterions of relevance and validity required for causal inference [4]. It is relevant for the strong significant associations of the instrumental variable with CSG-receipt in all first-stage regressions. It is valid as our various applied tests find strong support for the exclusion assumption to hold, e.g. the instrumental variable effects mental health only through CSG-receipt and the instrumental variable satisfies the conditional independence assumption. We present a more detailed discussion and the different applied tests in section *A2* of the Additional file 1.

As we use a binary instrumental variable for a binary endogenous variable, we effectively estimate the Wald-Estimator [4]. Using eligibility criteria as instrumental variable for programme participation is common in the literature [3]. The age-eligibility of a child has also been used as instrument for CSG participation at the individual-level in a previous analysis [12].

We use the parametric standard 2-Stage Least Squares (2SLS) estimator with both stages estimated simultaneously, first with pooled and then with FE specification and compare the results. FE 2SLS square estimation is feasible as both the instrumented and the instrumental variable vary over time. This approach has a clear strength as it allows accounting for time-invariant and time-variant unobserved individual heterogeneity simultaneously and is the preferred approach in this analysis [24].

The first stage can be formalised as follows:

3 $$ CS{G}_{i,t}={\gamma}_0+{\gamma}_1{Z}_{i,t}+{\boldsymbol{X}}_{i,t}{\gamma}_2+{\boldsymbol{T}}_{\boldsymbol{i}}{\gamma}_3+{\boldsymbol{R}}_{\boldsymbol{i}}{\gamma}_4+{a}_i+{u}_{i,t} $$

where *Z*_*i*, *t*_ is the instrumental variable. We use linear probability models throughout the estimation.

In the second stage we regress CES-D on the linear prediction of *CSG*_*i*, *t*_ from the first stage and the full set of covariates ***X***_*i*, *t*_.

4 $$ CE{S}_{i,t}={c}_0+{c}_1\hat{CS{G}_{i,t}}+{\boldsymbol{X}}_{i,t}{c}_2+{\boldsymbol{T}}_{\boldsymbol{i}}{c}_3+{\boldsymbol{R}}_{\boldsymbol{i}}{c}_4+{a}_i+{u}_{i,t} $$

*c*_1_is the effect of the instrumented *CSG*_*i*, *t*_ in the first stage estimation. As in equation (4.1), **X** is a vector of control variables, ***T***_***i***_ is a set of year dummies, ***R***_***i***_ is a set of regional dummy variables. We show in section *A2* that our instrumental variable approach satisfies the conditions to estimate the Average Treatment Effect on the Treated, which implies that results can be generalised to the full study population rather than to complying individuals [2].

### Heterogeneity of the effect by gender

We investigate heterogeneous effects by gender because there is evidence of gender differences in the effect of UCT on mental health [22]. We follow Kilburn et al. [22] and provide sub-sample estimates of our models to make them comparable with this study.

### Robustness checks

We carry out a number of robustness checks on the effects of attrition, on the threshold applied to identify poor (R800), on the assumption of sharing the cash grant within the household, a placebo-treatment receipt estimation to test anticipation effects and a test regressing mental health on child-eligibility using financially non-eligible households only. The last test is especially important as a non-significant coefficient for child-eligibility would support firstly the causality of cash transfer effects on mental health and secondly the assumption that child-age does not affect mental health directly (e.g. support the validity of the instrumental variable). We discuss the different robustness checks in more detail in section *A4* of the Additional file 1.

## Results

### Descriptive statistics

Table 1 provides descriptive statistics by wave for the 10,925 individuals in the full sample. The average respondent is in moderate to poor mental health. Approximately 75% of individuals live in households receiving CSG and about 87% live in a household that is eligible to receive the CSG. This is consistent with previous studies [8, 10, 12, 16, 18] and is evidence of potential selection into the CSG, as in about 50% of the non-recipient households there is at least one eligible child. Every third person in the sample is male. The average respondent is about 39 years old and lives with six other individuals together in a household in a moderately safe neighbourhood. About 13% of individuals in the sample are younger than 19 years of age. One in nine households has experienced the death of a household member in the past 2 years. 38% of the individuals report to have at least one individual in their household who receives the old age pension, 12% the disability grant, 5% the foster care grant and 1% the care dependency grant.

**Table 1:** Summary statistics 2008–2014

Variables	Definition	2008 ***n*** = 6801	2010 ***n*** = 7831	2012 ***n*** = 7818	2014 ***n*** = 6323	All years ***n*** = 10,925
***Outcome Variable***						
CES-D	Centre for Epidemiological Depression Scale ranging from 0 to 30; higher values indicate better mental health	18.99 (4.39)	20.07 (4.00)	20.12 (4.28)	20.36 (4.04)	19.90 (4.21)
***Main Explanatory Variable***						
Household CSG	1 if individual lives in a CSG- receiving household, 0 otherwise	0.65	0.76	0.80	0.83	0.76
***Instrumental variable***						
Household with CSG eligible child	1 if a child eligible for the CSG lives in the household, 0 otherwise (including no children)	0.83	0.87	0.90	0.89	0.87
***Covariates***						
Male	0 if female, 1 if male	0.32	0.34	0.32	0.29	0.32
Age	The age of the individual in years	37.58 (17.64)	37.86 (18.18)	38.61 (18.00)	40.96 (17.69)	38.68 (17.94)
Age under 19	1 if the individual is < 19 years of age, 0 otherwise	0.17	0.16	0.13	0.05	0.13
Econ. decision maker	1 if the individual is the economic decision maker of the household, 0 otherwise	0.39	0.39	0.41	0.46	0.41
Size of the household	The number of household members	5.91 (3.17)	6.62 (3.82)	6.60 (3.79)	6.55 (3.53)	6.43 (3.61)
Death in household	1 if a household member has died in the past 2 years, 0 otherwise	0.18	0.16	0.14	0.12	0.15
Household OAG	1 if individual lives in an Old Age Pension -receiving household, 0 otherwise	0.32	0.41	0.39	0.41	0.38
Household DG	1 if individual lives in a DG-receiving household, 0 otherwise	0.15	0.10	0.11	0.12	0.12
Household FCG	1 if individual lives in a FCG-receiving household, 0 otherwise	0.04	0.05	0.06	0.06	0.05
Household CDG	1 if individual lives in a CDG-receiving household, 0 otherwise	0.01	0.01	0.02	0.02	0.01
Neighbourhood Theft	0 “never happens in neighbourhood”, 1 “very rare in neighbourhood”, 2 “not common in neighbourhood”, 3 “fairly common in neighbourhood”, 4 “very common in neighbourhood”	1.88 (1.49)	1.84 (1.38)	2.26 (1.43)	2.21 (1.48)	2.05 (1.45)
***Region***	**The baseline is “Rural formal”**					
Tribal Authority Area	1 if the individual lives in a tribal authority area, 0 otherwise	0.53	0.54	0.56	0.55	0.54
Urban Formal	1 if the individual lives in an urban formal area, 0 otherwise	0.31	0.30	0.29	0.29	0.30
Urban Informal (Townships)	1 if the individual lives in an urban informal area, 0 otherwise	0.07	0.07	0.07	0.07	0.07
***Province***	**The baseline is “Limpopo”**					
Western Cape	1 if the individual lives in Western Cape, 0 otherwise	0.08	0.07	0.08	0.07	0.07
Eastern Cape	1 if the individual lives in Eastern Cape, 0 otherwise	0.14	0.14	0.14	0.14	0.14
Northern Cape	1 if the individual lives in Northern Cape, 0 otherwise	0.08	0.07	0.06	0.06	0.07
Free State	1 if the individual lives in Free State, 0 otherwise	0.06	0.05	0.05	0.05	0.06
KwaZulu-West	1 if the individual lives in KwaZulu-West, 0 otherwise	0.33	0.34	0.35	0.38	0.35
North West	1 if the individual lives in North West, 0 otherwise	0.08	0.08	0.07	0.06	0.07
Gauteng	1 if the individual lives in Gauteng, 0 otherwise	0.06	0.06	0.07	0.07	0.06
Mpumalanga	1 if the individual lives in Mpumalanga, 0 otherwise	0.07	0.07	0.07	0.07	0.07

Note: Descriptive statistics are on the sample of the estimated models. n indicates the number of individuals. Variable means (standard deviations when applicable)

Splitting the samples by waves shows the increase in the coverage of the CSG policy nationwide, with increasing numbers of individuals living in CSG receiving and/or in a CSG eligible household over time. Mental health improves slightly from an average of 19.0 points in 2008 to an average of 20.4 points in 2014. Table A2 discussed in section *A1* of the Additional file 1 compares individuals living in recipient and non-recipient households. We observe slightly higher CES-D scores for individuals living in receiving households (19.89 vs 19.37), similarities in other grant receipt and disparities in household composition with more household members, more female household members and lower mean age in receiving households. Statistical difference in means of control variables between individuals living in CSG-receiving households and non-recipient households suggest the potential absence of random selection of CSG-receipt.

### Pooled versus fixed effects

Table 2 reports the results of the pooled and FE estimation. We find a positive and significant effect on mental health for individuals living in a CSG receiving household across the models. In the pooled model without covariates in column (1), individuals living in a CSG receiving household have on average a 0.325 point higher CES-D score than individuals living in non-recipient households.

**Table 2:** Effect of CSG on mental health: Pooled OLS and Fixed Effect estimation

	(1)	(2)	(3)	(4)
	Pooled OLS without covariates	Pooled OLS with covariates	FE without covariates	FE with covariates
Household CSG	0.325***	0.405***	0.449***	0.536***
	(0.087)	(0.091)	(0.119)	(0.126)
Male		0.346***		
		(0.051)		
Age		−0.076***		0.136*
		(0.009)		(0.080)
Age Squared		0.001***		0.001
		(0.000)		(0.000)
Age under 19		0.513***		0.385**
		(0.097)		(0.169)
Economic decision maker		−0.143**		0.037
		(0.061)		(0.082)
Household Size		0.048**		0.045
		(0.022)		(0.038)
Negative Event		−0.215**		−0.048
		(0.094)		(0.116)
Neighbourhood Theft		−0.036		0.034
		(0.030)		(0.035)
Attrition		−0.194**		0.014
		(0.098)		(0.132)
Constant	18.778***	21.323***	18.759***	12.149***
	(0.122)	(0.324)	(0.131)	(3.031)
Year	YES	YES	YES	YES
Other Government Programmes	No	YES	No	YES
Child Age Dummy	No	YES	No	YES
Province	No	YES	No	YES
Region	No	YES	No	YES
Observations	28,773	28,773	28,773	28,773
Individuals			10,925	10,925
R-squared	0.016	0.066	0.020	0.025

The outcome variable is CES-D (0–30) the measure for depression; PSU clustered standard errors are in parenthesis; *** *p* < 0.01, ** *p* < 0.05, * *p* < 0.1

Controlling for all covariates in the pooled model in column (2) the coefficient increases to 0.405. This implies a 2% increase in CES-D score compared to the mean of CES-D. When controlling for unobserved heterogeneity in the models using FE without and with covariates in columns (3) and (4), results show that the size of the coefficient increases, and it remains statistically significant. This indicates a downward bias in the pooled OLS estimate due to correlation of the main explanatory variable with unobservable idiosyncratic factors. The coefficient size reported in column (4) is 0.536, which indicates a 2.7% increase in CES-D from the mean of CES-D.

In both the pooled and FE models, being younger than 19 years of age is associated with better mental health. In the FE model age has a positive non-linear association with mental health. Males have on average a significantly better mental health than females in the pooled model. In the pooled model, age has a non-linear, and positive above 30 years, association with mental health. In the pooled model, mental health is better in households of larger size. Economic decision making and negative events are both negatively associated with mental health.

Notably, attrition is significantly and negatively associated with transfer receipt in the pooled model in column (2). However, when using FE estimation this association loses statistical significance which tells us that FE address the correlation of attrition with unobservable factors determining mental health.

We perform a Hausman test to determine if FE or Random Effects models should be used. The Hausman test rejects the null hypothesis *p* < 0.001 in favour of the FE estimation.

### 2SLS and 2SLS FE estimation

The estimates of the first stage regression of the different instrumental variable models are presented in Table 3, for the whole population in columns (1) to (4) including pooled OLS without and with covariates and FE estimations without and with covariates, and FE estimation for male and female separately in columns (5) and (6). The regression of the household level CSG receipt on the instrumental variable in the top row of the table shows positive significant effects throughout all specifications.

**Table 3:** Effect of eligibility on CSG receipt: First Stage 2SLS, Fixed effect 2SLS estimation

	(1)	(2)	(3)	(4)	(5)	(6)
	First Stage 2SLS without covariates	First Stage 2SLS with covariates	First Stage FE without covariates	First Stage FE with covariates	First Stage FE Male	First Stage FE Female
CSG eligible child	0.833***	0.723***	0.712***	0.644***	0.636***	0.643***
	(0.006)	(0.011)	(0.014)	(0.016)	(0.022)	(0.017)
Male		−0.021***				
		(0.004)				
Age		0.001		0.011**	0.002	0.014**
		(0.001)		(0.005)	(0.011)	(0.006)
Age Squared		−0.000**		−0.000*	0.000	−0.000***
		(0.000)		(0.000)	(0.000)	(0.000)
Age under 19		−0.007		0.012	−0.003	0.017
		(0.007)		(0.011)	(0.018)	(0.014)
Economic decision maker		0.003		−0.001	−0.000	−0.002
		(0.005)		(0.005)	(0.012)	(0.007)
Household Size		−0.001		0.010***	0.014***	0.008***
		(0.003)		(0.003)	(0.004)	(0.003)
Negative Event		−0.010		0.005	0.002	0.007
		(0.008)		(0.009)	(0.014)	(0.009)
Neighbourhood Theft		0.003		−0.001	−0.002	0.000
		(0.002)		(0.002)	(0.003)	(0.003)
Attrition		−0.010		−0.005	0.013	−0.014
		(0.008)		(0.009)	(0.012)	(0.011)
Constant	−0.038***	− 0.040				
	(0.007)	(0.026)				
Year	YES	YES	YES	YES	YES	YES
Other Government Support	NO	YES	NO	YES	YES	YES
Child Age Dummy	NO	YES	NO	YES	YES	YES
Province	NO	YES	NO	YES	YES	YES
Region	NO	YES	NO	YES	YES	YES
Observations	28,773	28,773	28,773	28,773	9147	19,626
Individuals			10,925	10,925	3723	7202
R-squared	0.443	0.482	0.277	0.305	0.349	0.286

The outcome variable is a binary variable indicating if the individual lives in a CSG-receiving household or not; PSU clustered standard errors are in parenthesis; *** *p* < 0.01, ** *p* < 0.05, * *p* < 0.1

The magnitude of the coefficient of the instrumental variable varies over the specifications with highest magnitude for the 2SLS estimation without covariates (0.833). Adding covariates to the OLS estimation in model (2) reduces the coefficient to 0.723, indicating correlation of the instrumental variable with other factors. When using FE with 2SLS estimation without covariates, the magnitude is further reduced to 0.712, which indicates that FE account for unobservable constant factors. Adding covariates in model (4) to the FE estimation further reduces the magnitude of the coefficient to 0.644. The estimated coefficient gives the compliance rate for the group of transfer recipients with the instrumental variable. The compliance rate shown in model (1) is 83%, which implies that 83% of the recipients comply in their treatment status conditional on the instrumental variable.

Table 4 presents the findings of the second stage of the pooled and FE instrumental variable estimations for the same six models. The coefficient associated with the CSG is positive and significant in all models, except for the one estimated on the male sub-sample where it is not statistically significant (*p* = 0.392).

**Table 4:** Effect of CSG on mental health: Second Stage 2SLS, Fixed Effect 2SLS

	(1)	(2)	(3)	(4)	(5)	(6)
	2SLS without covariates	2SLS with covariates	FE 2SLS without covariates.	FE 2SLS with covariates	FE 2SLS Male	FE 2SLS Female
Household CSG	0.614***	0.749***	0.621***	0.822***	0.468	1.000***
	(0.125)	(0.152)	(0.231)	(0.279)	(0.447)	(0.323)
Male		0.368***				
		(0.051)				
Age		−0.077***		0.133*	0.087	0.146
		(0.009)		(0.080)	(0.146)	(0.092)
Age Squared		0.001***		0.001*	−0.001	0.001**
		(0.000)		(0.000)	(0.001)	(0.000)
Age under 19		0.517***		0.380**	0.734**	0.143
		(0.098)		(0.169)	(0.288)	(0.214)
Economic decision maker		−0.135**		0.041	−0.073	0.028
		(0.061)		(0.082)	(0.156)	(0.110)
Household Size		0.053**		0.042	0.014	0.057
		(0.022)		(0.037)	(0.067)	(0.039)
Negative Event		−0.208**		−0.050	0.386**	−0.215*
		(0.094)		(0.115)	(0.161)	(0.129)
Neighbourhood Theft		−0.036		0.034	0.043	0.032
		(0.030)		(0.035)	(0.047)	(0.038)
Attrition		−0.187*		0.015	−0.117	0.088
		(0.098)		(0.132)	(0.194)	(0.152)
Constant	18.588***	21.169***				
	(0.128)	(0.326)				
Year	YES	YES	YES	YES	YES	YES
Other Government Support	NO	YES	NO	YES	YES	YES
Child Age Dummy	NO	YES	NO	YES	YES	YES
Province	NO	YES	NO	YES	YES	YES
Region	NO	YES	NO	YES	YES	YES
Observations	28,773	28,773	28,773	28,773	9147	19,626
Individuals			10,925	10,925	3723	7202
R-squared	0.015	0.065	0.019	0.025	0.026	0.029

The outcome variable is CES-D (0–30) the measure for depression; PSU clustered standard errors are in parenthesis; *** *p* < 0.01, ** *p* < 0.05, * *p* < 0.1

In column (1) the effect of living in a receiving CSG household on mental health outcome, estimated using pooled 2SLS, increases mental health by about half a unit on the CES-D scale (0.614) compared to individuals living in non-receiving household. The magnitude of the effect is higher (0.749) in model (2) when controlling in the pooled 2SLS estimation for confounding factors in the determining mental health. The FE 2SLS estimation in column (3) shows a marginally larger magnitude compared to the 2SLS with a coefficient of size 0.621 which is a 15% standard deviation increase in mental health. The marginally larger size of the coefficient in column (3) compared to column (1) shows that the 2SLS estimation without covariates in (1) is downwardly biased due to unobserved heterogeneous factors.

Adding control variables in the FE 2SLS estimation in column (4) shows a transfer effect of size 0.822 on individuals’ mental health which is a 4.1% improvement in the mean value of mental health. The 2SLS coefficient of transfer receipt without FE shows low variation with and without conditioning on covariates whereas in the 2SLS estimation with FE it shows significant difference conditioning on covariates and without covariates. An explanation is that covariates are correlated with unobservable factors adding bias to the estimation, which we address by using individual FE model. The CSG effect is not significant for the male sub-sample in column (5) and the strongest cash transfer effect amongst all models is observed in the female sub-sample in column (6) with an improvement on the CES-D scale of one unit or an increase in mental health of 5 %.

The FE 2SLS model with covariates in column (4) is twice the size of the pooled model coefficient in column (2) of Table 2, indicating a potential downwardly bias of the pooled model-coefficient due to unobserved heterogeneity in mental health. The bias remains when controlling for unobserved heterogeneity in health but not instrumenting for the treatment receipt as the coefficient size of the FE in column (4) of Table 2 is still 0.3 points lower than the one estimated with FE 2SLS in column (4) of Table 4.

When controlling for determinants of mental health, the following coefficients estimated with the pooled 2SLS in column (2) are associated with mental health. We find positive significant effects for being under 19 years of age, being male and the size of the household. Significant negative associations for age (though with increasing positive non-linear age effects), economic decision making in a household, death of a household member and attrition. The attrition dummy is only significant in the pooled model. This indicates for the FE models that individuals who leave the survey for reasons of death or non-response are not systematically biasing the estimation.

We find in the FE model in column (4) a positive significant association of age with mental health. Both FE models in columns (4) and (5) show a positive effect of being under 19 years of age on mental health. The FE model for males in column (5) shows positive effects of negative events on male mental health. In contrast the FE model for females in column (6) identifies negative associations of negative events with mental health.

We test the statistical strength of the instrumental variable in all presented models. The results from these tests emphasise and support the statistical strength of our instrumental variable in all 2SLS models. In all first-stage regressions is the endogenous regressor neither weakly nor under-identified by the instrumental variable (F-statistics > 10) [41]. For brevity, we present in the following only results from the preferred FE 2SLS model in brackets, however the findings apply to all 2SLS models. Since only one instrumental variable is applied, the F-test, the Angrist-Pischke multivariate F test, and Kleibergen-Paap test are similar [4, 23]. The tests reject the null hypothesis at the 99% level (F = 633.76 Prob >F < 0.001). Since we can reject the null hypothesis, the instrument is strong and adequate [41]. Both the Stock-Wright and the Anderson-Rubin Wald test specifications tests reject the null hypothesis (Stock-Wright: *p* = 0.00212; Anderson-Rubin Wald test *p* = 0.0159), indicating that endogenous regressors are relevant. The critical value of the Stock-Yogo ID which is 16.38 and indicates a weak instrument threshold is far below the F-statistics (10% = 16.38 < 633.75) indicating a strong instrument [45]. The endogeneity test (Durbin-Wu-Hausman test or short Hausman form) is not rejected (t = 1.162, *p* = 0.2810). This tells us that self-selection is not based on mental health characteristics.

### Robustness checks

Our robustness checks show that the results are robust to sample selection and attrition, that mental health effects remain strong when actual cash transfer recipients are excluded from the sample estimation, which supports the argument of cash-transfer sharing on the household level, and that no placebo-effects of cash transfer receipt occur, supporting the claim that no anticipation effects occur. We find further strong support for the causality of cash transfer effects on mental health and the exclusion assumption (e.g. validity) of the instrumental variable to hold as living with a child in the CSG age-eligibility brackets in non-poor households (income above Rand800 and not applicable to apply for the CSG programme) has no statistically significant effect on mental health. An elaborate discussion of the findings from the robustness analysis is presented in section *A5* of the Additional file 1.

## Discussion

This is the first study to analyse the effect of a large unconditional cash transfer programme (UCT), the South African Child Support Grant, on mental health of adults, rather than specific population sub-groups, living in poverty. We use a large longitudinal sample of 10,925 individuals from four waves (2008–2014) of the National Income Dynamics Study, a representative survey of the South African population. We measure mental health with the Centre for Epidemiological Depression (CES-D) scale. Using a Fixed Effect instrumental variable approach to account for potential selection bias into the CSG, we find that the cash transfer has a strong positive effect on mental health of individuals living in recipient households by half a unit on a 30-point scale. We conducted sub-analyses by gender and identify a significant effect, only for females, twice the size of the male coefficient. Our results are robust to potential attrition, sample selection and placebo-effects. Our instrumental variable is relevant and statistically strong. Our various tests around the validity of the instrumental variable show all overwhelming evidence for the instrument’s validity to hold.

The finding of positive effects of a UCT on mental health fits into the behavioural economics and psycho-social literature on the relationship between poverty and mental health conditions [27]. It is also in line with firstly findings from studies analysing the short-term effects of UCTs on mental health on sub-populations from LMICs [5, 11, 20, 35] and secondly within the wider qualitative and quantitative literature on the effects of cash transfers on mental health in Sub-Saharan Africa [33].

Previous work on the effect of a UCT effect on adolescent mental health found different disparities with significant effects only for males [22]. The difference in findings can be explained by the different design of the cash transfer programmes. Kilburn et al. [22] used a UCT with a short-term horizon whereas the CSG is a long-term support programme. Differences can also occur due to the different sample populations. Kilburn et al. [22] used a sample of adolescents whereas our analysis contributed by using an adult sample. Our findings of significant effects fit into the epidemiological literature on the burden of mental health among the females. Female mental health is possibly more responsive to the UCT as women are at higher risk for common mental health disorders with 50% higher prevalence of depression for women [50].

The main challenge of this paper is that the programme is not randomly assigned. Our instrumental variable approach with Fixed Effects accounts for selection effects. The advantage of using a natural experiment like the CSG is the external validity of its implication for studying the mental health effects of cash transfer programmes. A limitation of instrumental variable estimation is the assumption that the instrumental variable only affects mental health through the endogenous variable and does not affect mental health directly and can be excluded from the mental health equation. This assumption untestable [4]. We found a range of evidence to support the validity of the exclusion assumption in this case. We found no effects of child-age on mental health, no effects using placebo-treatment estimations for individuals living with age-eligible children in households that are not financially eligible for the CSG.

We considered the use of alternative instrumental variables previously used in the literature as proxy for local government investment and access to government services, such as: distance to water sources from the house or dwelling, municipal expenditures in the past 30 days, household transport expenditures in the past 30 days, or the council level density of CSG receiving children, weighted by the number of council level residents.

None of these instruments were strong in predicting receipt of CSG, while our chosen instrumental variable, “household contains an eligible child”, is strong and valid in explaining cash transfer receipt. This instrumental variable varies exogenously over time because of the changes to the eligibility threshold making it stronger than a simple cross-section with a single eligibility criterion. Furthermore, we conducted several tests to support the validity and exclusion assumption of the instrumental variable.

We further presented strong arguments that our instrumental variable can be considered as good as randomly distributed as manipulation of the variable can be ruled out for the following reasons: firstly the exogeneous time-varying age-eligibility of the CSG, secondly empirical evidence finding no difference in odds for child birth between CSG-receiving an non receiving mothers, thirdly evidence suggesting the economic costs of bringing up children would be higher than the grant-income, and fourthly trends in fertility rates in South Africa remain unchanged after introduction of the CSG programme [16, 38, 42, 46].

We considered alternative estimation methods such as fuzzy regression discontinuity design using the age-eligibility cut-offs [25]. However, such an approach would not allow us to account for unobserved time invariant effects nor estimate population-wide mental health effects of UCTs as this method relies on comparing only a small subgroup of the population close to the cut-off point with very similar characteristics.

The aim of this study was to understand the effects of an UCT on mental health of the average poor adult population. Due to the design of the CSG, which requires a household to have an age-eligible child to receive the cash transfer, results may be representative of households with an eligible child, rather than a South African household. However, this is an unlikely concern for the composition of poor households in South Africa. In the average poor South African household live between three and four children, where children are defined as household members below age 18 [44].

The finding highlights that UCTs have strong and robust direct positive benefits for mental health of adult populations from LMICs. Following the Grossman model of health where more healthy time is an input factor of individual productivity [17], better mental health can enable individuals to improve their productivity which can then contribute to decrease poverty in the long-term. The effects of better mental health could possibly show strong spill-over effects on other dimensions, especially when considering the strong co-morbidities of mental with physical health and the strong relationship of mental health with poverty [28, 32].

## Conclusion

Our findings of strong positive effects of a UCT on mental health in South Africa have important implications for policies in LMICs. Firstly, UTCs have strong potential to reduce depressive symptoms among poor populations which is important in settings such as South Africa where unipolar depression is the main contributor among mental diseases to the overall burden of disease [21]. Secondly, in the South African context, population wide improvements in mental health could show significant effects on HIV-prevalence, due to the strong positive correlation between the two [29]. Thirdly, UCTs can be a relatively simple approach of dealing with the high burden of disease due to common mental health disorders such as depression in LMICs. They could have potential to overcome the big treatment gaps in mental health care by reducing the need for health care in settings where a substantial undersupply of trained health staff and mental health treatment facilities exists [29].

Whilst our research has established a positive effect of a UCT on mental health over a period of 6 years, with most individuals exposed to the UCT throughout that time, more research is required to understand long-term effects of UCTs on mental health beyond this period. Further should future research focus on understanding the heterogeneous effects of different amounts of UCTs on population-wide mental health.

## Supplementary information

**Additional file 1.**

## Data Availability

The NIDS data sets used for this study are publicly available at http://www.nids.uct.ac.za/nids-data/data-access.

## Abbreviations

CDG: Care Dependency Grant
CES-D: Centre for Epidemiological Depression Scale
CSG: Child Support Grant
DG: Disability Grant
FE: Fixed Effects
FCG: Foster Care Grant
NIDS: National Income Dynamics Study
OAP: Old Age Pension
UCT: Unconditional Cash Transfer

## References

[1] Allen J, Balfour R, Bell R, Marmot M (2014). Social determinants of mental health. Int Rev Psychiatry.

[2] Angrist JD (2004). Treatment effect heterogeneity in theory and practice. Econ J.

[3] Angrist JD, Krueger A (1991). Estimating the payoff to schooling using the Vietnam-era draft lottery. (NBER working paper).

[4] Angrist JD, Pischke J-S. Mostly harmless econometrics: an empiricist’s companion. Princeton: Princeton University Press; 2008.

[5] Baird S, de Hoop J, Ozler B, De Hoop J, Özler B (2013). Income shocks and adolescent mental health. J Hum Resour.

[6] Baron EC, Davies T, Lund C (2017). Validation of the 10-item Centre for Epidemiological Studies Depression Scale (CES-D-10) in Zulu, Xhosa and Afrikaans populations in South Africa. BMC Psychiatry.

[7] Clark AE (2007). Born to be mild? Cohort effects Don’t (fully) explain why well-being is U-shaped in age. (IZA discussion paper series).

[8] Cluver L, Boyes M, Orkin M, Pantelic M, Molwena T, Sherr L (2013). Child-focused state cash transfers and adolescent risk of HIV infection in South Africa: a propensity-score-matched case-control study. Lancet Glob Health.

[9] Das J, Do Q-T, Friedman J, McKenzie D, Scott K (2007). Mental health and poverty in developing countries: revisiting the relationship. Soc Sci Med.

[10] Delaney A, Ismail Z, Graham L, Ramkissoon Y (2008). Review of the child support Grant: uses, implementation and obstacles. (United Nations Children’s fund report).

[11] Eyal K, Burns J (2015). Up or down? Intergenerational mental health transmission and cash transfers in South Africa. (working paper).

[12] Eyal K, Woolard I (2011). Throwing the book at the CSG. (southern Africa labour and development research unit working paper series).

[13] Fernald LCH, Hidrobo M (2011). Effect of Ecuador’s cash transfer program (bono de Desarrollo Humano) on child development in infants and toddlers: a randomized effectiveness trial. Soc Sci Med.

[14] Gaarder MM, Glassman A, Todd JE (2010). Conditional cash transfers and health: unpacking the causal chain. J Dev Effect.

[15] Golberstein E, Busch S. Mental health, determinants of. In: Encyclopedia of health economics. 1st ed. Amsterdam: Elsevier; 2014.

[16] Gomersall J (2013). The performance of the child support Grant: review and research priorities. Dev South Afr.

[17] Grossman M (1972). Concept of health capital and demand for health. J Polit Econ.

[18] Hall K, Woolard I, Lake L, Smith C (2012). South African child gauge.

[19] Hauck K, Rice N (2004). A longitudinal analysis of mental health mobility in Britain. Health Econ.

[20] Haushofer J, Shapiro J (2016). The short-term impact of unconditional cash transfers to the poor: experimental evidence from Kenya. Q J Econ.

[21] Jack H, Wagner RG, Petersen I, Thom R, Newton CR, Stein A (2014). Closing the mental health treatment gap in South Africa: a review of costs and cost-effectiveness. Glob Health Action.

[22] Kilburn K, Thirumurthy H, Halpern CT, Pettifor A, Handa S (2016). Effects of a large-scale unconditional cash transfer program on mental health outcomes of young people in Kenya. J Adolesc Health.

[23] Kleibergen F, Paap R (2006). Generalized reduced rank tests using the singular value decomposition. J Econ.

[24] Le HT, Nguyen HT (2018). The impact of maternal mental health shocks on child health - estimates fromFixed-effects instrumental variables models for two cohorts of Australian children. Am J Health Econ.

[25] Lee DS, Lemieux T (2010). Regression discontinuity designs in economics. J Econ Lit.

[26] Leibbrandt M, Woolard I, De Villiers L (2009). Methodology: report on NIDS wave 1.

[27] Lund C (2012). Poverty and mental health: a review of practice and policies. Neuropsychiatry.

[28] Lund C, Breen A, Flisher AJ, Kakuma R, Corrigall J, Joska JA (2010). Poverty and common mental disorders in low and middle income countries: a systematic review. Soc Sci Med.

[29] Lund C, Petersen I, Kleintjes S, Bhana A (2012). Mental health Services in South Africa: taking stock. Afr J Psychiatry.

[30] Lund C, De Silva M, Plagerson S, Cooper S, Chisholm D, Das J (2011). Poverty and mental disorders: breaking the cycle in low-income and middle-income countries. Lancet.

[31] Mani A, Mullainathan S, Shafir E, Zhao J (2013). Poverty impedes cognitive function. Science.

[32] Ohrnberger J, Fichera E, Sutton M (2017). The dynamics of physical and mental health in the older population. J Econ Ageing.

[33] Owusu-Addo E, Renzaho AMN, Smith BJ (2018). The impact of cash transfers on social determinants of health and health inequalities in sub-Saharan Africa: a systematic. Health Policy Plan.

[34] Paxson C, Schady N (2010). Does money matter? The effects of cash transfers on child health and development in rural Ecuador. Econ Dev Cult Chang.

[35] Plagerson S, Patel V, Harpham T, Kielmann K, Mathee A (2011). Does money matter for mental health? Evidence from the child support Grants in Johannesburg, South Africa. Glob Public Health.

[36] Prince M, Patel V, Saxena S, Maj M, Maselko J, Phillips MR (2007). No health without mental health. Lancet.

[37] Radloff L (1977). The CES-D scale: a selt report depression scale for research in the general population. Appl Psychol Meas.

[38] Rosenberg M, Pettifor A, Nguyen N, Westreich D, Bor J, Bärnighausen T (2015). Relationship between receipt of a social protection grant for a child and second pregnancy rates among south African women: a cohort study. PLoS One.

[39] South African Government (2004). Social assistance act.

[40] Southern African Development Research Unit (2016). Wave 4 overview National Income Dynamics Study.

[41] Staiger BYD, Stock JH (1997). Instrumental variables regression with weak instruments. Econometrica.

[42] Statistics South Africa (2011). Fertility in South Africa - census 2011.

[43] Statistics South Africa (2014). Poverty trends in South Africa.

[44] Statistics South Africa. Poverty trends in South Africa: an examination of absolute poverty between 2006 and 2015. Pretoria: Statistics South Africa; 2017.

[45] Stock JH, Yogo M (2003). Testing for weak instruments in linear IV regression. (NBER working paper).

[46] UNICEF (2005). The state of the world’s children 2005 - childhood under threat. (United Nations Children’s fund report).

[47] UNICEF, DSD and SASSA (2016). Removing barriers to accessing child Grants.

[48] Verbeek M, Nijman T (1992). Testing for selectivity bias in panel data. Int Econ Rev.

[49] Wittenberg M. A comment on the use of “cluster” corrections in the context of panel data. Cape Town: National Income Dynamics Study; 2013.

[50] World Health Organization (2008). The global burden of disease: 2004 update.

[51] Wright PA, Kloos B (2007). Housing environment and mental health outcomes: analysis perspective. J Environ Psychol.

