# Supplementary material for: The effect of cash transfers on mental health – new evidence from South Africa
Source: BMC Public Health. 2020 Apr 3;20:436. doi: 10.1186/s12889-020-08596-7 (PMC7118950; doi:10.1186/s12889-020-08596-7)
Supplement: Supplementary file 1 — Additional file 1. [file 12889_2020_8596_MOESM1_ESM.docx]

**APPENDIX: *The effect of cash transfers on mental health – New Evidence from South Africa***

We present in section *A1* the descriptive statistics of individuals living in cash transfer receiving households with individuals living in cash transfer non-receiving households and descriptive statistics on individuals living in households variation in the instrumental (having an age-eligible child) versus those with no variation, in *A2* the supporting arguments and evidence for the validity and relevance conditions of the instrumental variable to hold, and the evidence for estimating the Average Treatment Effect on the Treated with the instrumental variable approach. We present in *A3* findings from the analysis on sample attrition and transition, in *A4* the empirical outline of the robustness checks and in *A5* the findings from the robustness checks.

***A1 Comparison of individuals by CSG status and instrumental variable***

Table A2 compares individuals living in recipient and non-recipient households. Individuals living in CSG recipient households have on average a slightly higher mean CES-D score (19.8 vs. 19.4) than individuals living in non-recipient households. About 42% of individuals from a non-recipient household live with a CSG eligible child, whereas 99.4% of individuals from a CSG receiving household live with a CSG eligible child. The composition of households varies by CSG status. In non-recipient households, 40% are male (24% in a receiving household), with average age of about 42 years (39 years in a receiving household). 57% of individuals in CSG-non-recipient household are involved in economic decision making compared to 49% in CSG-receiving households. The average size of recipient households is much larger (6 versus 3.5 individuals). The negative shock of a death in the household occurred to about 13% of individuals from non-recipient households compared to about 14% from receiving households. We observe a similar share of participation in the other social grant programmes between CSG recipient and non-recipient households.

Table A3 compares individuals at the first wave of the NIDS (2008) living in households in which the instrumental variable CSG age-eligible child in households shows variation over the panel period compared to individuals living in households with no variation in living with an age eligible child. We use this comparison as we estimate fixed effect models with instrumental variables, e.g. focus on within variation. We find overall good balance of characteristics between the two groups. Significant differences are observed in the variables males, age under 19, the household size, receipt of the Foster Care Grant, and living in Eastern Cape province.

***A2 Instrumental variable conditions and ATET***

*Instrumental variable conditions*

The instrumental variable must satisfy both the validity and the relevance conditions. Validity is satisfied when the exclusion restriction holds and the instrument affects the outcome variable only through the endogenous regressor conditional on confounders (Angrist and Krueger, 2001). While the exclusion assumption is not testable, we provide the following four arguments to support it.

Firstly, the instrumental variable is coded as one for all individuals if a child who is eligible to receive the CSG for his/her age lives in the household. This implies that individuals living with children of all other not eligible ages are coded as zero. This is crucial, as age differences around the CSG age cut-off threshold and changes of the CSG age cut –off over time are orthogonal to the outcome and unlikely to affect the outcome variable directly. For example, the age cut-off for CSG eligibility was 13 years in 2008. The assumption here is that having a child aged 13 or more does not impact mental health differently to having a child of age 12 years or less.

Secondly, we include a set of 19 binary variables of child age to the estimations. These 19 binary variables indicate if children of age [0,18] live in the household of the respondent and consequently will pick up any possible variation of the instrumental variable with mental health induced by each specific age of the cohabiting children. Thus, the instrumental variable should measure only the programme effects even when child-age mental health effects are present. Notably, in neither of the estimations where we control for child age effects, child effects are jointly significant in explaining adult mental health.

Thirdly, the CSG programme has time-varying age eligibility cut-offs. Due to this variation families with children of the same age have different exposures to the policy at different times. Thus, if child-age specific effects around cut-off points are present, these effects should balance out over the panel data as children of all ages become available to receive the CSG from 2012 onwards.

Fourthly, we identify no statistically significant effects of child eligibility on mental health of individuals living in CSG financially ineligible households (income >R800). This finding strongly supports the assumption that changes in mental health are caused by the cash transfers and not by differences in child age. If we drop the CSG receiving care taker from the analysis as he/she is supposedly the most affected person in the household by possible child-age related effects on mental health. The results remain strong and significant supporting our assumption that the estimated instrumental variable effects do not reflect age effects and that the instrumental variable is valid in identifying population effects.

The second condition, relevance, implies that the instrumental variable is correlated with the endogenous regressor and has to be uncorrelated with the error term (Angrist and Krueger, 2001). Firstly, correlation of the instrumental variable with the treatment variable at the first stage is satisfied in all first stage estimations. The correlation coefficient is always significant and shows strong magnitude in all estimations indicating that “household with a CSG eligible child” is a strong and relevant instrument for “household receives the CSG”.

Secondly, the instrumental variable is not correlated with unobservable factors of the first stage estimation and satisfies the conditional independence assumption. Factors that determine access to the CSG could possibly also affect the instrumental variable in the first stage estimation. We add control variables for potential confounders such as the region and province where the household lives. We use FE estimation which allows only for time-varying factors to affect the estimation. Thus, all parameters which are time-invariant such as individual preferences of adults for childbirth or individual preferences to apply for the CSG are taken out of the estimation. Furthermore, we follow Lee and Lemieux, (2010) and test the conditional independence of the instrumental variable by regressing each covariate on the instrumental variable whilst controlling for all other covariates using the individual fixed effect model. We present the estimates in table A6 and find that for all but one outcome (economic decision maker) has the instrumental variable no significant effect which is strong support for the conditional independence assumption.

Thirdly, the possible manipulation of the instrumental variable may affect the validity of the instrument leading to a bad instrument problem (Angrist and Krueger, 2001). In the context of the CSG this would be induced fertility among the poor population to access the CSG support. If manipulation occurs, the instrumental variable is no longer as if random and is instead determined by selection effects.

We rule out the manipulation of the instrumental variable for the following four reasons:

i.) Fertility rates across South Africa are continuously falling since 1996, prior to the onset of the programme. Fertility rates fall across age-groups, provinces and population and income groups. The latest fertility report of South Africa based on the census 2011 clearly shows that fertility declined between 1996 and 2011 from 3.23 children to 2.67 children (Statistics South Africa, 2011). More recent statistical evidence shows a further fall in fertility rates to 2.55 in 2015 (Statistics South Africa, 2015).

ii.) an empirical study on the effects of the CSG programme on fertility identified no difference in the odds for child birth between CSG receiving and non-receiving mothers which strongly supports the assumption of no programme induced perverse incentives of childbirth (Rosenberg et al., 2015).

iii.) data on the CSG and take-up rates suggest that especially in the first years of life of the new-born, take-up of the CSG programme is low (Cluver et al., 2013). If individuals were to have children for the sole reason to receiver transfer, take-up of the programme should be high in the first years for the economic cost arising from childbirth, and for missing out on the income flow.

iv.) estimates by the United Nations in 2005 show that the cost of raising a child from age 0-17 are about $16,000 on average for the poor population living in LMICs (UNICEF, 2005), which is about R108,540 (conversion rate 1:6.7 on average in 2005). The benefit of receiving the CSG in 2005 was R180 a month which over 17 years equals to R36,720, assuming that individuals did not anticipate increases in the monthly CSG rate. Assuming that the UN estimate is valid for South Africa, the cost of raising a child is 2.96 times as high as the benefit. This relative numbers suggest that the decision to have a child solely for the purpose of receiving the CSG grant is unlikely considering the higher cost.

A last concern of the relevance of the instrumental variable considers anticipation effects of programme receipt. Such effects can occur due to anticipation of future changes in the age-bandwidth for CSG eligibility or when household members are pregnant but not yet receiving the CSG support. To anticipate findings from the robustness analysis (placebo-estimation) with respect to this concern, we can exclude that such effects bias the instrumental variable.

Following these arguments, we have strong evidence to exclude bias due to manipulation of our instrumental variable. This supports the relevance of the instrumental variable.

*Estimating the Average Treatment Effect on the Treated*

A key assumption of instrumental variable estimation is that individual preferences for treatment are monotonic, implying that defiers are excluded (Angrist and Pischke, 2008). When relevance, validity and monotonicity are satisfied, we estimate the Local Average Treatment Effect (LATE), which is the effect for the sub-population of treatment compliers (Angrist and Pischke, 2008). In a special case, when always-taking behaviour can be excluded, the LATE simplifies to the Average Treatment Effect on the Treated (ATET) which implies that results can be generalised to the full study population rather than to complying individuals (Angrist, 2004). This special case applies to this study as the share of “Always takers” is negligible (see table A4).

***A3 Attrition and transition***

Table A5 describes attrition and transition between the waves. Transition is defined here as moving in or out of the sample study due to increase or reduction in income and falling below or stepping above the R800 income threshold. Between 2008 and 2010, 1,135 of 6,801 individuals moved above the income threshold with 593 individuals moving below the threshold. Transition increases in both directions over the years involving 2,355 of 7,818 individuals moving above the threshold between 2012 and 2014 and 1,011 moving below the threshold. Attrition is defined here as temporarily or permanently leaving the survey between the waves. 4,215 individuals left the study between 2008 and 2010, 4,715 between 2010 and 2012 and 2,678 between 2012 and 2014. Attrition amongst poor individuals amounts to 2,769 individuals between 2008 and 2010, 3,271 between 2010 and 2012, and 2,678 from 2012 to 2014.

Mental health outcomes are similar for individuals moving out of the lower income bracket and individuals moving into the lower income bracket (19.69 vs. 19.65 over 2008-2010, 20.42 vs. 20.17 over 2010-2012, 20.63 vs. 20.88 over 2012-2014. The average CES-D amongst the individuals leaving the survey between the waves is fairly similar to the average of the survey samples for the waves. Individuals leaving between 2008 and 2010 had a mean CES-D score of 19.44 vs. 18.99 for the study sample in 2008, individuals leaving between 2010 and 2012 had a mean CES-D score of 20.319 vs. 20.07 of observations in 2010 (table 2a), and 20.633 versus 20.12 in 2012 (table 2a). The mean scores of CES-D amongst low-income individuals leaving the survey between waves are fairly similar to the sample by each wave.

***A4 Robustness checks***

*Sample Selection*

Livelihoods of individuals can improve over time, for example due to the cash transfer. As a result, they can move out of poverty over the course of the study. This group of individuals is included in the full NIDS survey but excluded from my analysis. Considering the assumptions of cash support programmes, those individuals close to the monetary eligibility threshold at the onset of the survey are likely to fall into this group (and so are the new entries which are marginally above the threshold at the onset). Ignoring potential sample selection induced by improved income for households receiving cash transfer payment can lead to a downward bias in the estimates.

Therefore, we run four sub-analyses using the instrumental variable approach with fixed effect estimation to test for sample selection. In the first sub-analysis, we keep only eligible individuals from wave one and estimate the model for all years using this reduced sample. This can give us an idea if my findings are robust to drop-outs enforced by applying the selected threshold. In a second approach, we estimate the model with the full sample, including all income groups to test robustness of the cash transfer effects on mental health across the full population. Finally, we use the full balanced panel sample irrespective of income group and in the fourth model we use the balanced sample of my preferred study sample. The third and fourth sub-analyses address concerns regarding attrition as balanced panel samples suffer most from selection bias, if a bias is present (Wooldridge, 2001).

*Excluding the recipient of the cash transfer*

A core assumption of the study is that cash transfers received by care takers are shared on a household level. We test this assumption by re-estimating our models excluding those individuals who are cash transfer recipient within the household. We would expect the coefficient associated with receiving CSG to be unchanged if cash transfers were shared within the household.

*Placebo treatment estimation*

Another robustness check addresses the differences in characteristics of households receiving and non-receiving the cash transfer. We run a set of placebo-estimations to understand if these conditional variations matter and how independent the effect of the cash transfer is for mental health. We use a reduced sample size in this analysis composed as follows: at the time of the comparison, in wave 1-3 neither household receives the cash transfer but a sub-set of households will receive the transfer in wave 4. We use a dummy variable which identifies individuals living in households that will receive the cash transfer at wave 4 but who have not received the CSG in any previous wave as “1” and “0” otherwise. We compare never receivers with receivers only in the final wave, wave four, but estimate this model using data on the balanced sample on the first three waves. We use cross-sectional IV as my dummy variable would drop from the estimation in a fixed effects model because of the lack of variation. Individuals in households receiving CSG in the final wave are coded in wave ones to three as one, while the never-receivers are coded as zero.

We undertake three estimations, first a pooled cross-sectional analysis over the first three waves, second an instrumental variable analysis over the first three waves and finally an instrumental variable analysis over all four waves. The instrument is the binary variable indicating if the individual lives in a household with a child of CSG eligible age. Non-significance of the placebo-dummy in the first and second estimation would imply that the cash transfer and the instrumented cash transfer are truly exogenous to household characteristics and affects mental health whilst controlling for confounders. Significance in the third estimation would further support the causal effect of the cash transfer on mental health outcomes. Non-significance would also imply that no anticipation effect for the cash transfer programme occurs, which would further support the validity of the instrumental variable.

*Using non-eligible to test treatment causality*

A last robustness test is directed at the causality of the cash transfer effect on mental health. We use a “placebo” sample of individuals living in household that are not-eligible to receive the CSG because household income is above the threshold of Rand800 per capita. We use all four waves in a fixed effect estimation of mental health on our instrumental binary variable “individual lives with a CSG age eligible child” while controlling for covariates. A statistically significant effect of the dummy variable on mental health would imply that the age of the child indeed affects mental health directly even though households are not eligible to receive CSG. No statistically significant effect would support the assumption that CSG affects mental health because of the cash transfer and not because of the age of the child.

***A5 Findings from the robustness checks***

In Tables A7 and A8 we present the analysis of sample selection effects and attrition. In table A9 and table A10 we present the analysis of the cash transfer effect on mental health without the care taking recipient(s) in the household and the placebo analysis of instrumented and non-instrumented cash transfer receipt. Table A11 presents a last supporting test on the exclusion assumption of the instrumental variable.

The robustness analysis regarding the sample composition (table A7 and A8) shows that the selection model is robust to changes in the margin and to transition in income, as the coefficient associated with being a CSG recipient remains statistically significant and of similar magnitudes in all models. When estimating the IV model on the balanced panel, the results are qualitatively unaffected, indicating again that attrition due to death or non-response does not bias my sample estimation.

Table A9 presents the results from the first stage of the 2SLS estimation on the sample excluding the individual in CSG-recipient households in columns (1), of the placebo-sample test over the first three waves in column (2) and of the placebo sample with all four waves in column (3). The instrumental variable is valid for the reduced sample in column (1) and not significant in column (2). This indicates that the instrumental variable is tracking changes and variations in CSG recipients correctly as none of the individuals receive the CSG over the first three waves. Using the placebo sample on all four waves, the instrumental variable is significant as expected.

In table A10, estimates of the second stage of the sample excluding the care taking individuals receiving the CSG-recipient(s) in model (1) show a significant strong effect of the instrumental variable on mental health (1.220). This finding supports the argument that the cash transfer is shared within the household among the household members and not only kept and used by the actual recipient(s). In model (2), which shows the results of the first three waves pooled cross-sectional OLS regression on the actual binary “last wave CSG”, no significant effect is associated with being future CSG recipients and mental health. No significant effect is observed in model (3), using the instrumental variable approach over the first three waves. In model (4), we find a strong significant positive effect (6.5) of the cash transfer on mental health when the placebo-recipients actually receive the cash transfer in the final round compared to the never-recipients. These findings support the causality of the cash transfer effect on mental health and the validity of the instrumental variable against possible anticipation effects.

Table A11 presents the estimation of the effect of mental health on child CSG age eligibility, using the all waves with individuals living in financially non-eligible households. The binary instrumental variable indicating if an individual lives with a CSG age eligible child has no significant effect on mental health. This further supports our claim of no effects of child age eligibility on adult mental health and overall the exclusion assumption of the chosen instrumental variable.

APPENDIX: Tables

Table A1 Child Support Grant age and income eligibility criteria and grant value between 1998 and 2014

| **Legislation date** | **Eligible age (years)** | **Income threshold (South African Rand per month)** | **Grant amount (South African Rand per month)** |
| --- | --- | --- | --- |
| 01/10/1998 | 0-7 | R 800 in Rural Areas;  R 1,100 in Urban Areas  Unchanged until October 2008 | R 100 |
| 01/07/1999 | 0-7 |  | R 100 |
| 01/07/2000 | 0-7 |  | R 100 |
| 01/07/2001 | 0-7 |  | R 110 |
| 01/04/2002 | 0-7 |  | R 140 |
| 01/10/2002 | 0-7 |  | R 160 |
| 01/04/2003 | 0-9 |  | R 160 |
| 01/04/2004 | 0-11 |  | R 170 |
| 01/04/2005 | 0-14 |  | R 180 |
| 01/04/2006 | 0-14 |  | R 190 |
| 01/04/2007 | 0-14 |  | R 200 |
| 01/04/2008 | 0-14 |  | R 210 |
| 01/10/2008 | 0-14 | R 2,300 | R 230 |
| 01/01/2009 | 0-15 | R 2,400 | R 240 |
| 01/04/2010 | 0-16 | R 2,500 | R 250 |
| 01/04/2011 | 0-17 | R 2,600 | R 260 |
| 01/01/2012 | 0-18 | R 2,800 | R 280 |
| 01/04/2013 | 0-18 | R 2,900 | R 290 |
| 01/04/2014 | 0-18 | R 3,100 | R 310 |
| 01/10/2014 | 0-18 | R 3,200 | R 320 |

Source: (Eyal and Burns, 2015): Notes: A.) Age refers to the upper age limit. B.) The income threshold for CSG eligibility was defined as 10 times the grant amount in October 2008 to adjust for constantly increasing price-inflation. C.) If the primary caregiver is married, the income threshold is doubled, for instance to R 6,400 per month in October 2014.

Table A2 Summary statistics comparison of individuals in household receiving and non-receiving CSG

|  | **CSG Non-Receiving Household (n=3,855)** | **CSG-Receiving Household (n=9,682)** | **t-test of difference** |
| --- | --- | --- | --- |
| CES-D | 19.373 (4.042) | 19.887 (3.705) | -7.099 |
| Household with CSG eligible child | 0.426 | 0.994 | -109.816 |
| Male | 0.393 | 0.236 | 26.160 |
| Age (years) | 41.867 (14.749) | 39.125 (12.273) | 11.055 |
| Age under 19 | 0.090 | 0.105 | -3.950 |
| Economic decision maker | 0.569 | 0.490 | 11.413 |
| Size of the household | 3.513 (2.013) | 6.084 (2.897) | -50.485 |
| Death in household | 0.130 | 0.144 | -2.027 |
| Household Old Age Pension | 0.307 | 0.331 | -2.756 |
| Household DG | 0.097 | 0.107 | -1.745 |
| Household FCG | 0.048 | 0.043 | 1.215 |
| Household CDG | 0.014 | 0.012 | 1.127 |
| Neighbourhood Theft | 2.035 (1.457) | 2.066 (1.454) | -1.111 |
| Region | 1.44 (0.795) | 1.337 (0.734) | 7.572 |
| Attrition | 0.114 | 0.090 | 5.136 |
| *Note: Descriptive statistics are on the sample of the estimated models over all years. n indicates the number of households. Variable means (standard deviations). T-test are testing for Ho: diff = 0 in mean.* | | | |

Table A3 Summary statistics: comparison of individuals in households with and without variation in age eligible children in household (instrumental variable), baseline 2008

|  | **(1) Households: No panel variation of child eligibility** | **(2) Households: Panel variation of child eligibility** | **t-test of difference in means** |
| --- | --- | --- | --- |
| CES-D | 18.884 (3.868) | 18.704 (3.843) | 1.099 |
| Male | **0.262** | **0.352** | **-6.562** |
| Age (years) | 38.779 (12.857) | 39.115 (14.024) | -0.605 |
| Age under 19 | **0.129** | **0.163** | **-3.368** |
| Economic decision maker | 0.478 | 0.461 | 1.065 |
| Size of the household | **5.275 (2.705)** | **4.068 (2.433)** | **10.757** |
| Death in household | 0.166 | 0.156 | 0.663 |
| Household Old Age Pension | 0.286 | 0.297 | -0.599 |
| Household DG | 0.126 | 0.143 | -1.187 |
| Household FCG | **0.040** | **0.024** | **2.045** |
| Household CDG | 0.009 | 0.010 | -0.209 |
| Neighbourhood Theft | 1.867 (1.479) | 1.864 (1.536) | 0.042 |
| *Province* |  |  |  |
| Limpopo | 0.090 | 0.095 | -0.410 |
| Western Cape | 0.091 | 0.078 | 1.123 |
| Eastern Cape | **0.146** | **0.185** | **-2.573** |
| Northern Cape | 0.082 | 0.062 | 1.732 |
| Free State | 0.066 | 0.083 | -1.657 |
| KwaZulu-West | 0.317 | 0.303 | 0.721 |
| North West | 0.069 | 0.058 | 1.079 |
| Gauteng | 0.067 | 0.079 | -1.157 |
| Mpumalanga | 0.072 | 0.057 | 1.476 |
| *Region* |  |  |  |
| Rural Formal | 0.095 | 0.086 | 0.684 |
| Tribal Authority | 0.505 | 0.526 | -1.014 |
| Urban Formal | 0.326 | 0.314 | 0.583 |
| Urban Informal | 0.075 | 0.074 | 0.136 |
| Note: Descriptive statistics compare individuals by household living with changing status of the instrumental variable over the four NIDS waves to individuals by households with no change. This is effectively the instrumental variable in the fixed effect estimation. We present here the sample of the estimated models at NIDS baseline 2008. The number of households in (1) are 2,651 and in (2) 707. Variable means (standard deviations). T-test are testing for Ho: diff = 0 in mean values. Bold values indicate significant differences of means (p-val <0.05). | | | |

**Table A4 Number of compliers, always takers, never takers of the CSG on the household level by NIDS waves**

|  | **2008** | **2010** | **2012** | **2014** |
| --- | --- | --- | --- | --- |
| Compliers | 5,546 | 6,828 | 6,943 | 4,913 |
| Always takers | 0 | 41 | 24 | 22 |
| Never takers | 1,189 | 918 | 748 | 340 |
| Share of Always takers with compliers | 0.000 | 0.006 | 0.003 | 0.005 |
| Share of Never takers with compliers | 0.214 | 0.134 | 0.107 | 0.069 |

*Defiers are ruled out by the monotonicity assumption of the LATE saying that individuals have a clear preference over their choice to participate in the grant scheme.

Table A5 Summary statistics transition and attrition between NIDS waves

|  | **2008-2010** | **2010-2012** | **2012-2014** |
| --- | --- | --- | --- |
| $t+1>R800\geq t$ | 1,135 | 1,710 | 2,355 |
| $t+1\leq R800<t$ | 593 | 604 | 1,011 |
| Attrition | 4,215 | 4,715 | 4,197 |
| Attrition $R\leq800$ | 2,769 | 3,271 | 2,678 |
| CES-D $t+1>R800\geq t$ | 19.692 (0.138) | 20.423 (0.102) | 20.634 (0.090) |
| CES $t+1\leq R800<t$ | 19.657 (0.196) | 20.178 (0.181) | 20.889 (0.141) |
| CES-D Attrition | 19.44 (0.071) | 20.319 (0.060) | 20.633 (0.072) |
| CES Attrition $R\leq800$ | 18.902 (0.085) | 20.115 (0.072) | 20.428 (0.086) |

Mean values reported for CES-D. Standard deviations in parenthesis.

Table A6 Testing Conditional independence of the Instrumental variable with covariates as outcomes and controls

|  | (1) | (2) | (3) | (4) | (5) | (6) | (7) | (8) | (9) | (10) |
| --- | --- | --- | --- | --- | --- | --- | --- | --- | --- | --- |
|  | **Age** | **Age under 19** | **Economic decision maker** | **HH Size** | **Negative Event** | **Old Age Pension** | **Disability Grant** | **Foster Grant** | **Care Dep. Grant** | **Neighbourhood Theft** |
|  |  |  |  |  |  |  |  |  |  |  |
| CSG eligible child | -0.007 | 0.006 | -0.059*** | -0.049 | -0.006 | 0.018 | -0.007 | 0.010 | 0.005 | -0.094 |
|  | (0.018) | (0.008) | (0.013) | (0.089) | (0.018) | (0.016) | (0.016) | (0.008) | (0.004) | (0.068) |
| Constant | 35.507*** | 1.015*** | 0.915*** | 3.004** | 0.011 | 0.036 | 0.064 | -0.270** | 0.027 | 2.591*** |
|  | (0.082) | (0.149) | (0.253) | (1.302) | (0.198) | (0.165) | (0.148) | (0.130) | (0.063) | (0.904) |
|  |  |  |  |  |  |  |  |  |  |  |
| Year | YES | YES | YES | YES | YES | YES | YES | YES | YES | YES |
| Control variables | YES | YES | YES | YES | YES | YES | YES | YES | YES | YES |
| Child Age Dummy | YES | YES | YES | YES | YES | YES | YES | YES | YES | YES |
| Region | YES | YES | YES | YES | YES | YES | YES | YES | YES | YES |
| Province | YES | YES | YES | YES | YES | YES | YES | YES | YES | YES |
| Observations | 28,773 | 28,773 | 28,773 | 28,773 | 28,773 | 28,773 | 28,773 | 28,773 | 28,773 | 28,773 |
| Individuals | 10,925 | 10,925 | 10,925 | 10,925 | 10,925 | 10,925 | 10,925 | 10,925 | 10,925 | 10,925 |
| R-squared | 0.968 | 0.225 | 0.066 | 0.651 | 0.021 | 0.102 | 0.056 | 0.017 | 0.017 | 0.032 |
| The outcome variables are indicated in the top row. All models use individuals fixed effects and control for the full set of covariates apart from the outcome covariate. We estimate linear models for (1), (4), and (10) and for all others linear probability models; PSU clustered standard errors are in parenthesis; *** p<0.01, ** p<0.05, * p<0.1. | | | | | | | | | | |

Table A7 Robustness tests: Sample estimations: First stage

|  | (1) | (2) | (3) | (4) | (5) |
| --- | --- | --- | --- | --- | --- |
|  | Study Sample | Baseline | Full Sample | Full Balanced | Study Balanced |
| CSG eligible child | 0.644*** | 0.596*** | 0.544*** | 0.546*** | 0.636*** |
|  | (0.016) | (0.014) | (0.014) | (0.016) | (0.018) |
| Age | 0.011** | 0.016*** | 0.013*** | 0.017*** | 0.014** |
|  | (0.005) | (0.004) | (0.004) | (0.004) | (0.007) |
| Age Squared | -0.000* | -0.000*** | -0.000*** | -0.000** | -0.000 |
|  | (0.000) | (0.000) | (0.000) | (0.000) | (0.000) |
| Age under 19 | 0.012 | 0.031*** | 0.014 | 0.015 | 0.011 |
|  | (0.011) | (0.011) | (0.009) | (0.015) | (0.018) |
| Economic decision maker | -0.001 | -0.000 | -0.001 | -0.001 | -0.003 |
|  | (0.005) | (0.005) | (0.004) | (0.005) | (0.007) |
| Household Size | 0.010*** | 0.012*** | 0.012*** | 0.013*** | 0.010*** |
|  | (0.003) | (0.003) | (0.003) | (0.003) | (0.003) |
| Negative Event | 0.005 | 0.002 | 0.010 | 0.006 | 0.006 |
|  | (0.009) | (0.009) | (0.008) | (0.009) | (0.010) |
| Neighbourhood Theft | -0.001 | 0.000 | 0.001 | -0.000 | -0.004 |
|  | (0.002) | (0.002) | (0.002) | (0.002) | (0.003) |
| Attrition | -0.005 | 0.002 | -0.003 |  |  |
|  | (0.009) | (0.009) | (0.006) |  |  |
| Year | YES | YES | YES | YES | YES |
| Other Government Support | YES | YES | YES | YES | YES |
| Child Age Dummy | YES | YES | YES | YES | YES |
| Province | YES | YES | YES | YES | YES |
| Region | YES | YES | YES | YES | YES |
| Observations | 28,773 | 29,614 | 48,872 | 26,553 | 16,573 |
| Individuals | 10,925 | 9,567 | 17,164 | 7,717 | 5,540 |
| R-squared | 0.305 | 0.345 | 0.326 | 0.322 | 0.303 |
| The outcome variable is the binary indicating if the individual lives in a household that received the CSG or not; (1) is the sample applied throughout this study, with restricting the sample to per capita income <=R800, (2) is the using individuals recorded in the baseline according to the selection in (1) throughout the waves, (3) is the full NIDS sample, (4) is the full balanced NIDS sample, (5) is the balanced sample (1). We control for the full set of covariates and where indicated for year, other government support programmes, child age, province and region effects; PSU clustered standard errors are in parenthesis; *** p<0.01, ** p<0.05, * p<0.1. | | | | | |

Table A8 Robustness tests: Sample estimations: Second stage

|  | (1) | (2) | (3) | (4) | (5) |
| --- | --- | --- | --- | --- | --- |
|  | Study Sample | Baseline | Full Sample | Full Balanced | Study Balanced |
|  |  |  |  |  |  |
| Household CSG | 0.822*** | 0.673*** | 0.502** | 0.861*** | 1.147*** |
|  | (0.279) | (0.234) | (0.214) | (0.255) | (0.300) |
| Age | 0.133* | -0.001 | 0.083 | 0.003 | 0.002 |
|  | (0.080) | (0.077) | (0.070) | (0.079) | (0.098) |
| Age Squared | 0.001* | 0.001** | 0.000 | 0.001* | 0.001** |
|  | (0.000) | (0.000) | (0.000) | (0.000) | (0.000) |
| Age under 19 | 0.380** | 0.681*** | 0.464*** | 0.691*** | 0.445* |
|  | (0.169) | (0.173) | (0.135) | (0.199) | (0.234) |
| Economic decision maker | 0.041 | 0.103 | 0.000 | -0.005 | 0.033 |
|  | (0.082) | (0.075) | (0.059) | (0.074) | (0.097) |
| Household Size | 0.042 | 0.056* | 0.054* | 0.059* | 0.066* |
|  | (0.037) | (0.032) | (0.030) | (0.032) | (0.036) |
| Negative Event | -0.050 | -0.213* | -0.189* | -0.349*** | -0.277** |
|  | (0.115) | (0.109) | (0.099) | (0.112) | (0.123) |
| Neighbourhood Theft | 0.034 | -0.005 | 0.021 | 0.001 | 0.006 |
|  | (0.035) | (0.031) | (0.027) | (0.031) | (0.041) |
| Attrition | 0.015 | -0.118 | -0.100 |  |  |
|  | (0.132) | (0.128) | (0.098) |  |  |
|  |  |  |  |  |  |
| Year | YES | YES | YES | YES | YES |
| Other Government Support | YES | YES | YES | YES | YES |
| Child Age Dummy | YES | YES | YES | YES | YES |
| Province | YES | YES | YES | YES | YES |
| Region | YES | YES | YES | YES | YES |
| Observations | 28,773 | 29,614 | 48,872 | 26,553 | 16,573 |
| Individuals | 10,925 | 9,567 | 17,164 | 7,717 | 5,540 |
| R-squared | 0.025 | 0.021 | 0.015 | 0.019 | 0.028 |
| The outcome variable is CES-D (0-30) the measure for depression;(1) is the sample applied throughout this study, with restricting the sample to per capita income <=R800, (2) is the using individuals recorded in the baseline according to the selection in (1) throughout the waves, (3) is the full NIDS sample, (4) is the full balanced NIDS sample, (5) is the balanced sample (1). We control for the full set of covariates and where indicated for year, other government support programmes, child age, province and region effects; PSU clustered standard errors are in parenthesis; *** p<0.01, ** p<0.05, * p<0.1. | | | | | |

Table A8 Robustness test: Sample estimations: Placebo tests and non-recipient test first stage

|  | (1) | (2) | (3) |
| --- | --- | --- | --- |
|  | First Stage:  HH without  immediate CSG Recipient | First Stage:  Placebo test  wave 1-3 | First Stage:  Placebo test  all waves |
| CSG eligible child | 0.534*** | 0.045 | 0.135*** |
|  | (0.019) | (0.065) | (0.023) |
| Male |  | -0.088*** | 0.007 |
|  |  | (0.030) | (0.009) |
| Age | -0.005 | -0.008 | 0.002 |
|  | (0.007) | (0.005) | (0.002) |
| Age Squared | 0.000** | 0.000 | -0.000 |
|  | (0.000) | (0.000) | (0.000) |
| Age under 19 | 0.037** | -0.057 | -0.001 |
|  | (0.015) | (0.052) | (0.017) |
| Economic decision maker | -0.021** | 0.011 | -0.009 |
|  | (0.009) | (0.026) | (0.009) |
| Household Size | 0.025*** | 0.057*** | 0.013*** |
|  | (0.005) | (0.015) | (0.005) |
| Negative Event | 0.004 | -0.013 | 0.019 |
|  | (0.012) | (0.032) | (0.013) |
| Neighbourhood Theft | -0.002 | -0.002 | -0.003 |
|  | (0.003) | (0.010) | (0.004) |
| Attrition | -0.008 |  |  |
|  | (0.011) |  |  |
| Constant |  | 0.515*** | -0.078 |
|  |  | (0.167) | (0.053) |
|  |  |  |  |
| Year | YES | YES | YES |
| Other Government Support | YES | YES | YES |
| Child Age Dummy | YES | YES | YES |
| Province | YES | YES | YES |
| Region | YES | YES | YES |
| Observations | 16,332 | 1,749 | 2,122 |
| Individuals | 6,808 |  |  |
| R-squared | 0.315 | 0.144 | 0.486 |
| The outcome variable in (1) is the variable indicating if the individual lives in a CSG-receiving household", in (2)and (3) is the binary variable indicating if the individual lives in a household which receives the CSG only in the last wave; Model (1) is the first stage instrumental variable estimation of the study sample without the receiving care taker of the grant in the household, (2) is the pooled cross-sectional instrumental variable first stage estimation of the placebo test comparing individuals living in never-receiving households with individuals that live in a household which receives the CSG only in the last wave, a balanced panel over the first three waves is used for the estimation, (3) is taking all four waves for the instrumental variable pooled-cross sectional analysis into account but the comparison remains. We control for the full set of covariates and where indicated for year, other government support programmes, child age, province and region effects; PSU clustered standard errors are in parenthesis; *** p<0.01, ** p<0.05, * p<0.1 | | | |

Table A10 Robustness test: Sample estimations: Placebo tests and non-recipient test including second stage

|  | (1) | (2) | (3) | (4) |
| --- | --- | --- | --- | --- |
|  | FE 2SLS:  without immediate CSG Recipient | Placebo test wave 1-3 | 2SLS:  Placebo test wave 1-3 | 2SLS:  Placebo test all waves |
| Last Wave CSG |  | 0.104 | 22.370 |  |
|  |  | (0.266) | (31.447) |  |
| Household CSG | 1.220*** |  |  | 7.016*** |
|  | (0.388) |  |  | (2.485) |
| Male |  | 0.482** | 2.480 | 0.620*** |
|  |  | (0.196) | (2.877) | (0.195) |
| Age | 0.025 | -0.082** | 0.086 | -0.086*** |
|  | (0.109) | (0.034) | (0.270) | (0.034) |
| Age Squared | 0.001 | 0.001** | -0.001 | 0.001** |
|  | (0.001) | (0.000) | (0.002) | (0.000) |
| Age under 19 | 0.256 | 0.406 | 1.736 | 0.642 |
|  | (0.220) | (0.535) | (2.307) | (0.525) |
| Economic decision maker | 0.044 | -0.455** | -0.698 | -0.322 |
|  | (0.122) | (0.230) | (0.700) | (0.206) |
| Household Size | -0.041 | 0.035 | -1.246 | -0.002 |
|  | (0.051) | (0.097) | (1.868) | (0.103) |
| Negative Event | 0.089 | -0.241 | 0.043 | -0.507* |
|  | (0.139) | (0.304) | (0.931) | (0.294) |
| Neighbourhood Theft | 0.055 | -0.131 | -0.080 | -0.098 |
|  | (0.041) | (0.080) | (0.254) | (0.078) |
| Attrition | 0.142 |  |  |  |
|  | (0.156) |  |  |  |
| Constant |  | 22.142*** | 10.621 | 22.136*** |
|  |  | (1.002) | (16.709) | (1.029) |
| Year | YES | YES | YES | YES |
| Other Government Support | YES | YES | YES | YES |
| Child Age Dummy | YES | YES | YES | YES |
| Province | YES | YES | YES | YES |
| Region | YES | YES | YES | YES |
| Observations | 16,332 | 1,749 | 1,749 | 2,122 |
| Individuals | 6,808 |  |  |  |
| R-squared | 0.019 | 0.108 | -4.248 | 0.025 |
| The outcome variable is CES-D (0-30) the measure for depression; (1) is the instrumental variable estimation of the study sample without the receiving care taker of the grant in the household, (2) is the pooled cross-sectional estimation of the placebo test comparing individuals living in never-receiving households with individuals that live in a household which receives the CSG only in the last wave, a balanced panel over the first three waves is used for the estimation, (3) is the same as in (2) but using the cross-sectional pooled instrumental variable “eligible child for CSG in household” for the placebo test, (4) is taking all four waves for the instrumental variable pooled-cross sectional analysis into account but the comparison remains. We control for the full set of covariates and where indicated for year, other government support programmes, child age, province and region effects; PSU clustered standard errors are in parenthesis; *** p<0.01, ** p<0.05, * p<0.1. | | | | |

Table A11 Fixed effect estimation of CES-D on child eligibility with individuals living in financially non-eligible households

|  | (1) |
| --- | --- |
|  | FE  with covariates |
|  |  |
| CSG eligible child | 0.204 |
|  | (0.257) |
| Age | 0.093 |
|  | (0.152) |
| Age Squared | -0.000 |
|  | (0.001) |
| Age under 19 | 0.787** |
|  | (0.334) |
| Economic decision maker | -0.021 |
|  | (0.125) |
| Household Size | 0.011 |
|  | (0.084) |
| Negative Event | -0.502* |
|  | (0.274) |
| Neighbourhood Theft | 0.043 |
|  | (0.044) |
| Attrition | -0.243 |
|  | (0.178) |
| Constant | 17.371*** |
|  | (5.160) |
|  |  |
| Year | YES |
| Other Government Support | YES |
| Child Age Dummy | YES |
| Province | YES |
| Region | YES |
| Observations | 21,720 |
| Individuals | 13,982 |
| R-squared | 0.016 |
| The outcome variable is CES-D (0-30) the measure for depression. We control for the full set of covariates; PSU clustered standard errors are in parenthesis; *** p<0.01, ** p<0.05, * p<0.1. | |

**APPENDIX: References**

Angrist, J. D. (2004) ‘Treatment Effect Heterogeneity in Theory and Practice.’ *The Economic Journal*, 114(2002) pp. 52–84.

Angrist, J. D. and Krueger, A. B. (2001) ‘Instrumental Variables and the Search for Identification: From Supply and Demand to Natural Experiments.’ *Journal of Economic Perspectives*, 15(4) pp. 69–85.

Angrist, J. D. and Pischke, J.-S. (2008) *Mostly harmless econometrics : An empiricist’s companion*. Princeton University Press.

Cluver, L., Boyes, M., Orkin, M., Pantelic, M., Molwena, T. and Sherr, L. (2013) ‘Child-focused state cash transfers and adolescent risk of HIV infection in South Africa: a propensity-score-matched case-control study.’ *The Lancet Global Health*, 1(6) pp. 362–370.

Eyal, K. and Burns, J. (2015) *Up or Down? Intergenerational Mental Health Transmission and Cash Transfers in South Africa*. (Working Paper).

Lee, D. S. and Lemieux, T. (2010) ‘Regression Discontinuity Designs in Economics.’ *Journal of Economic Literature*, 48(2) pp. 281–355.

Rosenberg, M., Pettifor, A., Nguyen, N., Westreich, D., Bor, J., Bï¿½rnighausen, T., Mee, P., Twine, R., Tollman, S. and Kahn, K. (2015) ‘Relationship between receipt of a social protection grant for a child and second pregnancy rates among South African women: A cohort study.’ *PLoS ONE*, 10(9) pp. 1–12.

Statistics South Africa (2011) *Fertility in South Africa - Census 2011*.

Statistics South Africa (2015) *Mid-year population estimates 2015*.

UNICEF (2005) *The State of the World ’ S Children 2005 - Childhood Under Threat*. (United Nations Children’s Fund Report).

Wooldridge, J. M. (2001) *Econometric Analysis of Cross Section and Panel Data*. *The MIT Press*.
